# Outcomes of HIV-associated pneumocystis pneumonia at a South African referral hospital

**DOI:** 10.1371/journal.pone.0201733

**Published:** 2018-08-02

**Authors:** Nondumiso Chiliza, Mariette Du Toit, Sean Wasserman

**Affiliations:** 1 Department of Medicine, Faculty of Health Sciences, University of Cape Town, Cape Town, South Africa; 2 Wellcome Centre for Infectious Diseases Research in Africa, Institute of Infectious Disease and Molecular Medicine, Division of Infectious Diseases and HIV Medicine, University of Cape Town, Cape Town, South Africa; University of Minnesota, UNITED STATES

## Abstract

HIV-associated pneumocystis pneumonia (PCP) is increasingly recognized as an important cause of severe respiratory illness in sub-Saharan Africa. Outcomes of HIV-infected patients with PCP, especially those requiring intensive care unit (ICU) admission, have not been adequately studied in sub-Saharan Africa. The aim of this study was to describe the clinical phenotype and outcomes of HIV-associated PCP in a group of hospitalized South African patients, and to identify predictors of mortality. We conducted a retrospective record review at an academic referral center in Cape Town. HIV-infected patients over the age of 18 years with definite (any positive laboratory test) or probable PCP (defined according to the WHO/CDC clinical case definition) were included. The primary outcome measure was 90-day mortality. Logistic regression and Cox proportional hazards models were constructed to identify factors associated with mortality. We screened 562 test requests between 1 May 2004 and 31 April 2015; 124 PCP cases (68 confirmed and 56 probable) were included in the analysis. Median age was 34 years (interquartile range, IQR, 29 to 41), 89 (72%) were female, and median CD4 cell count was 26 cells/mm3 (IQR 12 to 70). Patients admitted to the ICU (n = 42) had more severe impairment of gas exchange (median ratio of arterial to inspired oxygen (PaO2:FiO2) 158 mmHg vs. 243 mmHg, p < 0.0001), and increased markers of systemic inflammation compared to those admitted to the ward (n = 82). Twenty-nine (23.6%) patients were newly-diagnosed with tuberculosis during their admission. Twenty-six (61.9%) patients admitted to ICU and 21 (25.9%) admitted to the ward had died at 90-days post-admission. Significant predictors of 90-day mortality included PaO2:FiO2 ratio (aOR 3.7; 95% CI, 1.1 to 12.9 for every 50 mgHg decrease), serum LDH (aOR 2.1; 95% CI, 1.1 to 4.1 for every 500 U/L increase), and concomitant antituberculosis therapy (aOR 82; 95% CI, 1.9 to 3525.4; P = 0.021). PaO2:FiO2 < 100 mmHg was significantly associated with inpatient death (aHR 3.8; 95% CI, 1.6 to 8.9; P = 0.003). HIV-associated PCP was associated with a severe clinical phenotype and high rates of tuberculosis co-infection. Mortality was high, particularly in patients admitted to the ICU, but was comparable to other settings. Prognostic indictors could be used to inform ICU admission policy for patients with this condition.

## Introduction

HIV-associated pneumocystis pneumonia (PCP) is increasingly recognized as an important cause of severe respiratory illness in sub-Saharan Africa [1]. A recent systematic review found that PCP may account for almost a quarter of cases of community-acquired pneumonia amongst inpatients in this region, with a case fatality of over 18% [2]. In the early part of the AIDS epidemic PCP was a common reason for intensive care unit (ICU) admission in the United States [3], and outcomes were extremely poor when patients required mechanical ventilation [4–6]. Some centers excluded patients with a diagnosis of PCP from ICU admission [7] because of the poor prognosis associated with respiratory failure. However, this practice has been recently challenged by a number of studies showing improved ICU outcomes with modern ventilation strategies [8] and the provision of ART [9], with survival ranging from 58% to 74% in high-income countries post-1996 [8, 10–12]. Outcomes of HIV-infected patients with PCP, especially those requiring ICU admission, have not been adequately studied in sub-Saharan Africa [1, 13, 14], where biological differences and limited access to care may influence disease outcomes [1]. This knowledge is important to identify higher risk patients and guide appropriate allocation of scarce resources, such as ICU beds. The aim of this study was to describe the clinical phenotype and outcomes of HIV-associated PCP in a group of hospitalized South African patients, and to identify predictors of mortality in this population.

## Methods

### Setting

We conducted a retrospective record review of HIV-associated PCP cases at Groote Schuur Hospital, a large academic referral center in Cape Town. The hospital operates four general medical wards with 128 beds offering secondary level and tertiary care, a combined medical and surgical ICU comprising 25 beds, and a 16-bedded high care unit for patients not requiring invasive ventilation. The ICU is staffed by Critical Care specialists; it admits patients directly from the Emergency Unit or medical wards and accepts transfers of complicated cases from peripheral hospitals.

### Inclusion criteria and search strategy

Cases were identified through an electronic search of the National Health Laboratory Service (NHLS) data warehouse. The search included all requests for PCP diagnostic testing on respiratory specimens from patients ≥ 18 years of age admitted to the hospital from April 2004. This was the earliest date that electronic laboratory reports were available and was also time of introduction of antiretroviral therapy into the public health service. We screened the medical records of all patients with any positive test result, all patients with negative results from ICUs and high care units, as well as those listed as being HIV-infected or having suspected PCP on the laboratory request forms. HIV-infected patients over the age of 18 years with definite or probable PCP were included in the analysis. Definite PCP was defined as the detection of *P jirovecii* cysts on immunofluorescent microscopy (the laboratory method used throughout the study period). The definition for probable PCP was based on the World Health Organization clinical case definition [15], and required the presence of appropriate clinical features (dyspnoea or cough of recent onset, tachypnoea, fever), suggestive radiographic abnormalities (evidence of diffuse bilateral interstitial infiltrates on chest X-ray), and the decision by clinicians to initiate empiric treatment for PCP. This information was extracted from medical records for all screened patients with a negative laboratory test for *P jirovecii*.

### Data collection

Baseline demographic and clinical data were extracted from medical records, including details relating to the presentation and severity of the index episode of PCP. Details of the hospitalization course were also obtained, including ICU admission and the use of mechanical ventilation, medical therapy, and initiation of antiretroviral therapy (ART) prior to discharge. We recorded results of routine laboratory testing as well as specific diagnostic tests performed for PCP and other respiratory infections, including tuberculosis. These were obtained from the NHLS electronic database. Other clinical outcomes included the occurrence of adverse reactions to PCP therapy, length of hospital stay, and survival to hospital discharge. Vital status and discharge data were obtained from the electronic Provincial patient tracking system, Clinicom. All data were anonymized, entered onto hardcopy case report forms and then captured into a specifically-designed electronic database. The database was checked independently by a separate investigator for omissions and inconsistencies with the paper forms.

### Analysis

Summary statistics were used to describe the demographic and clinical characteristics of PCP cases, stratified by ICU admission. Comparisons between the groups were performed using Wilcoxon rank-sum tests for non-parametric continuous variables and Chi-squared tests for dichotomous variables. The proportion (and 95% confidence intervals, CI) of patients with inpatient and 90-day mortality was calculated and compared by ICU admission using Chi-squared testing and crude and adjusted odds ratios. Univariate logistic regression analysis was performed to identify factors associated with this outcome. We tested the following *a priori* variables, which were thought to be clinically relevant or had been identified as predictors of PCP mortality in previous studies: age,[8, 12] sex,[16] CD4 cell count, knowledge of HIV diagnosis prior to presentation with PCP,[8] receipt of cotrimoxazole prophylaxis,[17] previous or current tuberculosis, confirmed PCP diagnosis, initiation of ART during admission,[9] the ratio of arterial to inspired oxygen (PaO2:FiO2 ratio) at presentation,[6] haemoglobin,[18] serum lactate dehydrogenase (LDH),[19] and serum albumin.[6, 9] Because this study aimed to explore predictors of outcomes in our particular setting, we also did a *post hoc* examination of factors which were found to be significantly different between patients admitted to ICU and the general wards. Variables with a P-value cutoff of < 0.1 on univariate analysis were included in a multivariate logistic regression model, which was performed using a stepwise forward selection procedure. Independent continuous variables were also presented in clinically-relevant categories. Predictors that resulted in increased variance without an impact on coefficient size were dropped from the final model to avoid collinearity. The maximum number of variables retained in the final model was restricted by the number of outcome events to a maximum of one variable per ten events.[20] Adjusted odds ratios and 95% confidence intervals were calculated. The Hosmer-Lemeshow statistic was used to assess the calibration of the final model; discriminative ability was quantified by the area under the receiver operating characteristic (ROC) curve. Internal validation of the model was performed using 100 bootstrap re-samples.

We also performed a survival analysis for 30-day mortality and compared the crude and adjusted survivor functions using the log rank test; these were plotted on Kaplan-Meier graphs. A multivariate Cox model was constructed to adjust for confounding predictors, using a similar approach to that described above for the binary outcomes, and results are reported as hazard ratios (HR) with 95% CIs. Proportional hazards assumptions were evaluated by performing a global test on Schoenfield residuals. Stata software version 14.2 (StataCorp) was used for statistical analysis.

This study was approved by the University of Cape Town Human Research Ethics Committee (ref 548/2015), who waived the requirement for informed consent for this retrospective study.

## Results

### Patient characteristics

**Fig 1:**
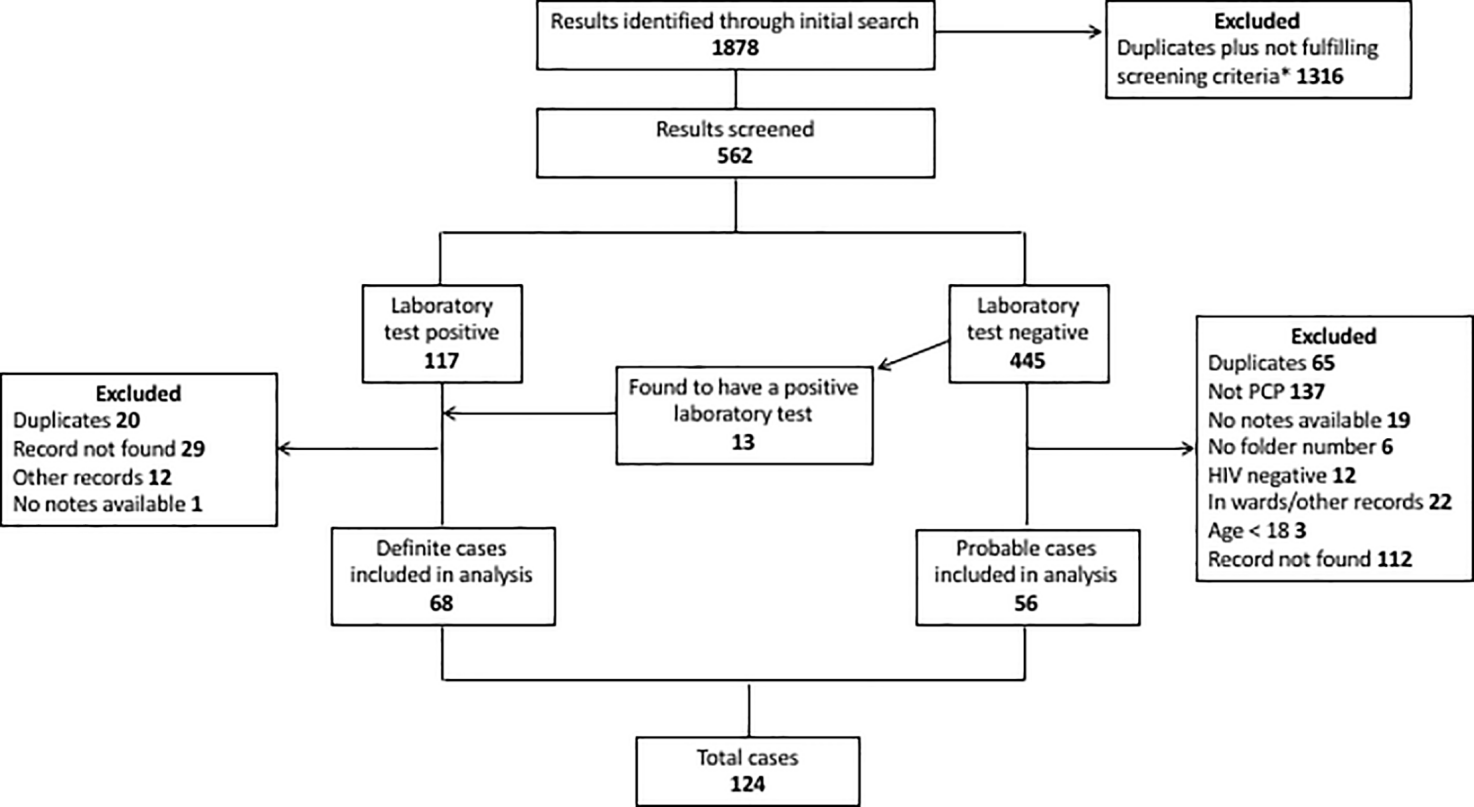
Consort diagram. * Criteria for screening records included: any positive test result, all patients with negative results from ICUs and high care units, laboratory request forms listing HIV infection or suspected PCP.

We screened 562 test requests between 1 May 2004 and 31 April 2015. A total of 124 PCP cases (68 confirmed and 56 probable) were included in the analysis after review of eligibility criteria and exclusion of duplicates (Fig 1). Baseline characteristics, stratified by ICU admission, are shown in Table 1. Patients admitted to ICU had a shorter duration of symptoms (7 vs. 14 days, p = 0.0008), more severe impairment of gas exchange (PaO2:FiO2 ratio 158 mmHg (IQR 116–218) vs. 243 mmHg (IQR 180–282), p < 0.0001), and increased markers of systemic inflammation compared to those admitted to the ward. Fewer patients admitted to ICU had a pre-existing diagnosis of HIV (38% vs. 66%, p = 0.003), which may reflect a bias due to restrictive admission criteria for suspected PCP. The most frequent presenting symptoms included cough (85%), dyspnoea (46%), weight loss (54%), sputum production (48%), night sweats (26%), chest pain (22%), and fever (19%).

**Table 1:** Baseline characteristics.

Variable	ICU (n = 42)	Ward (n = 82)
Age, years (range)	32 (19–62)	36.5 (20–62)
Female	31 (74)	58 (71)
Weight, kg	68 (45–84)	55.5 (50–63)
HIV diagnosed during index admission^a^	26 (62)**	27 (34)
ART-naive	36 (86)	67 (82)
CD4 cell count, cells/μL^b^	26 (10–67)	27 (12–73)
Definite PCP	23 (55)	45 (55)
Cotrimoxazole prophylaxis^c^	2 (13)	12 (25)
Previous TB^d^	10 (24)	35 (43)*
Duration of symptoms, days^e^	7 (6–14)**	14 (12–28)
Respiratory rate, breaths/min^d^	40 (30–55)	37 (32–40)
Systolic blood pressure, mm/Hg	94 (107–120)	110 (94–123)
Pulse rate, beats/min	128 (118–140)**	104 (117–133)
Oxygen saturation, %^f^	81 (70–87)**	87 (79–92)
Partial pressure of arterial oxygen, kPa^g^	6.8 (5.8–7.4)	7.2 (5.7–8.0)
Arterial to inspired oxygen (PaO2:FiO2) ratio, mmHg^h^	158 (116–218)***	243 (180–282)
White cell count, 10^9^/L^d^	13.4 (8.6–16.7)***	7.2 (5.4–10.5)
Haemoglobin, g/dL^d^	9.7 (9.1–12.3)	10.6 (9.5–12.5)
Serum albumin, mg/dL^i^	22 (18–26)***	28.5 (24–33)
Serum creatinine, μmol/L^d^	77 (51–147)	64 (52–84)
C-reactive protein, mg/dL^j^	117 (87–147)	72 (48–142)
Lactate dehydrogenase, U/L^k^	1639 (767–2231)	987 (733–1554)

Data are n (%) or median (IQR).

* P < 0.05

** p < 0.01

*** p < 0.0001.

Denominators are

a. n = 121

b. n = 117

c. n = 64

d. n = 123

e. n = 104

f. n = 94

g. n = 79

h. n = 101

i. n = 75

j. n = 42

k. n = 66

### Management and course

Induced sputum (n = 90, 73.2%) was the most common diagnostic specimen sent for PCP testing, of which 45 (50%) were positive. The majority of induced sputum samples (n = 78, 87%) were collected from patients managed in the ward. The next most frequent specimen in this population was tracheal aspiration (n = 27, 21.9%), of which 17 (63%) were positive. Tracheal aspirates were performed on ICU patients only. PCP treatment was started within two days of admission in 75% of cases, although this ranged from 0 to 36 days. All patients received cotrimoxazole as initial therapy, but 8 (6%) required a switch to alternative therapy as a result of adverse drug reactions. Initial intravenous cotrimoxazole was administered in 24 (20%) patients, and 16 (20%) patients were switched from oral to intravenous therapy during the admission. Corticosteroids were prescribed in 114 (92%) patients overall. Ten (12%) ward patients did not receive corticosteroids, possibly because they were not sufficiently hypoxic (mean PaO2:FiO2 ratio 309 mmHg versus 229 mmHg in those who received steroids, P = 0.04). Antiretroviral therapy was initiated in 7 (6.8%) ART-naive inpatients. Overall, 102 (82.2%) of patients received concomitant antibiotics, and 5 (4%) were prescribed oseltamivir. Median length of hospital stay for ward patients was 9 days (IQR 6 to 15), which was shorter than for those admitted to the ICU (13 days, IQR 8 to 19, P = 0.01).

Twenty-nine (23.6%; 17 with definite PCP) patients were newly-diagnosed with tuberculosis during their admission, three with Kaposi sarcoma, and two with cryptococcal disease. Sputum microscopy and culture was positive for bacteria in 14 patients (n = 98, 14.3%; 8 with definite PCP), mainly with Gram-negative pathogens.

ICU management is summarized in Table 2. The longest delay to ICU admission after presentation to hospital was 3 days, and all but six patients were admitted to ICU on the day of their arrival. The median duration of stay in the ICU was 7 days (IQR 5 to 15, range 1 to 33), and was not different between survivors and non-survivors (P = 0.482).

**Table 2:** Management of ICU patients with PCP.

Variable	ICU (n = 42)
Duration of stay, days	7 (5–15)
Mechanical ventilation	41 (98)
Initial FiO2, %	60 (60–70)
Initial positive end-expiratory pressure, cmH_2_0	10 (8–10)
Duration of mechanical ventilation, days	6 (4–14, range)
Haemodynamic support	20 (47)
Duration of haemodynamic support, days	2 (1–4)
Ventilator-associated pneumonia	20 (47)
Pneumothorax	1 (2)

Data are n (%) or median (IQR)

### PCP outcomes

The number of deaths occurring in hospital and at 90 days post admission are summarized in Table 3. Ninety-day mortality occurred in 26 (61.9%) of those admitted to ICU and in 21 (25.9%) of admitted to the ward (unadjusted odds ratio (OR) for ICU admission, 4.6; 95% CI, 2.1 to 10.3; P < 0.0001). ICU admission did not remain an independent predictor of inpatient (P = 0.112) or 90-day (P = 0.143) mortality when adjusted for differences in baseline characteristics, although a trend with similar magnitude persisted (data not shown).

**Table 3:** Outcomes.

Variable	ICU (n = 42)	Ward (n = 82)	Overall (n = 124)
90-day mortality	26 (61.9)	21 (25.9)	47 (38.2)
Inpatient mortality	24 (57.1)	15 (18.3)	39 (31.5)

Data are n(%)

In addition to ICU admission, significant predictors of 90-day mortality in univariate analysis included low PaO2:FiO2 ratio (OR 1.01; 95% CI, 1.0 to 1.02) and serum albumin (OR 1.1; 95% CI, 1.0 to 1.2). Unknown HIV diagnosis on admission (OR 1.9; 95% CI, 0.9 to 4.1), concomitant antituberculosis therapy (OR 2.1; 95% CI, 0.87 to 4.9), and serum LDH (OR 1.0; 95% CI, 0.99 to 1.0) showed borderline significance and were also included in the multivariable analysis. ICU admission was not included in the multivariate model because of multicollinearity. In the final model PaO2:FiO2 ratio, serum LDH, and concomitant antituberculosis therapy remained independent predictors of 90-day mortality. Concomitant antituberculosis therapy had a large effect size, but this had poor precision (adjusted OR 82; 95% CI, 1.9 to 3525.4; P = 0.021). Although the effect size per unit decrease of PaO2:FiO2 ratio appears negligible (aOR 1.03; 95% CI, 1.00 to 1.1), this translates into a 3.7-fold (95% CI, 1.1 to 12.9) increased odds of mortality for every 50 mmHg increase in the ratio. Similarly, for serum LDH every 500 U/L increase results in increased odds (aOR 2.1; 95% CI, 1.1 to 4.1) of death at 90 days.

The Hosmer–Lemeshow test χ^2^ statistic P-value was 0.536, suggesting adequate model fit, and discriminative ability was reasonable with a ROC area of 0.87 (95% CI, 0.76 to 0.99). The equivalent AUC of the ROC in bootstrap validation was 0.81 (95% CI, 0.67 to 0.95), yielding an optimism estimate of 0.0661. Results from logistic regression are shown in Table 4.

**Table 4:** Predictors of 90-day mortality.

Univariate			Multivariate	
Variable	OR (95% CI)	P-value	OR (95% CI)	P-value
PaO2:FiO2 ratio (per 50 mmHg decrease)	1.7 (1.2 to 2.4)	0.001	3.7 (1.1 to 12.9)	0.039
Serum albumin (per unit decrease)	1.1 (1.0 to 1.2)	0.021	1.0 (0.9 to 1.2)	0.614
Unknown HIV diagnosis	1.9 (0.9 to 4.1)	0.082	1.6 (0.3 to 9.8)	0.627
Serum LDH (per 500 U/L increase	1.3 (0.7 to 1.7)	0.085	3.4 (1.2 to 9.8)	0.025
Current TB treatment	2.1 (0.9 to 4.9)	0.102	82 (1.9 to 3525.4)	0.021

The following variables were also tested in univariate analysis, but had P-values > 0.1: age, sex, CD4 cell count, receipt of cotrimoxazole prophylaxis, previous tuberculosis, confirmed PCP diagnosis, initiation of ART during admission, duration of symptoms, pulse, white blood cell count.

**Fig 2:**
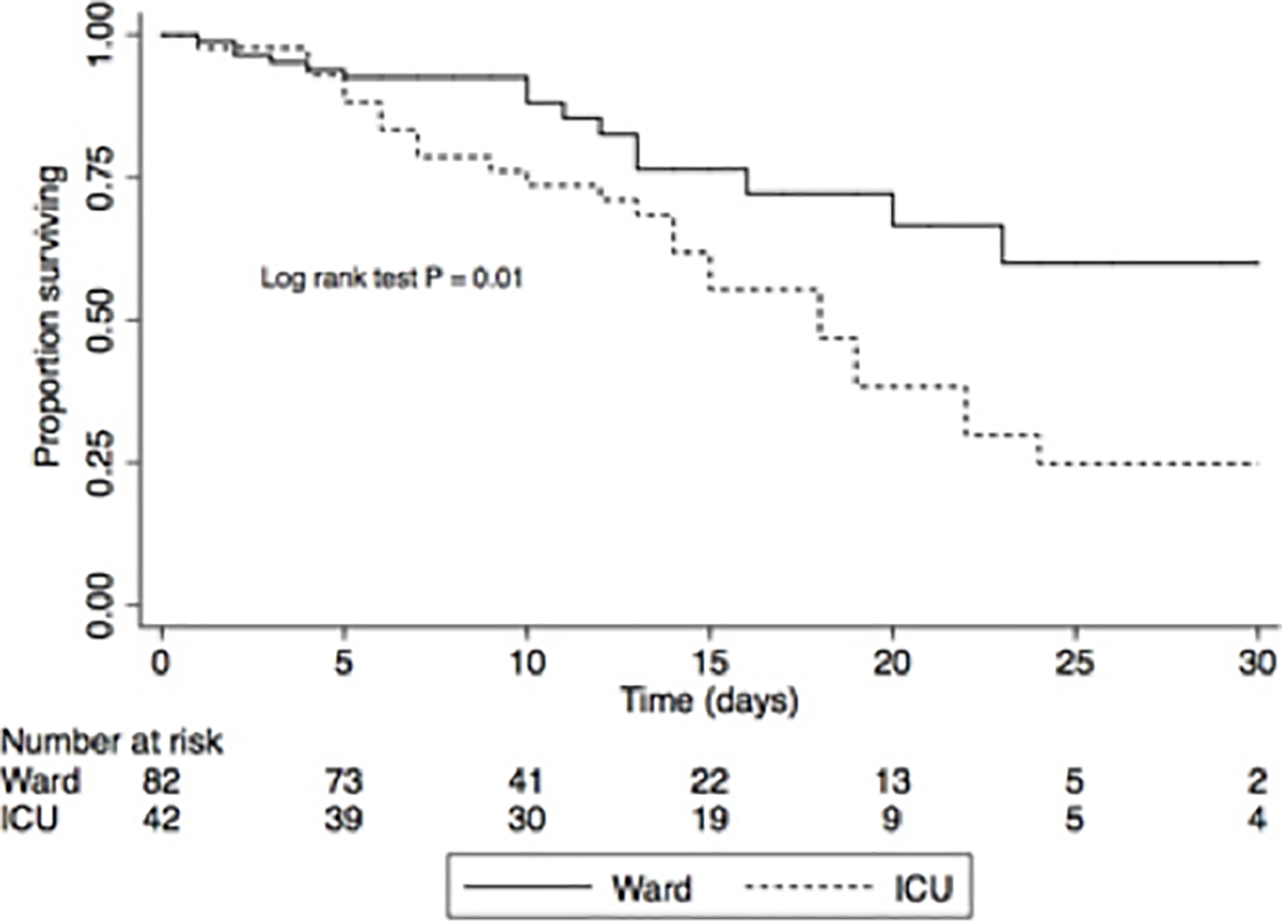
Crude survival estimates stratified by ICU admission.

Unadjusted 30-day survival estimates, stratified by ICU admission, are shown in Fig 2. The crude hazard ratio (HR) for ICU versus ward mortality was 2.3 (95% CI 1.2 to 4.3, P = 0.014). The median time to death amongst ICU patients was 13.5 days versus 9.5 days for those admitted to the ward. Serum LDH (HR 1.0; 95% CI, 1.0 to 1.0; P = 0.009), PaO2:FiO2 ratio (HR 1.01; 95% CI, 0.99 to 1.01; P = 0.054), and current TB treatment (HR 1.8; 95% CI, 0.9 to 3.5; P = 0.086) were identified as significant predictors on univariate Cox regression. LDH was not included in the final multivariate model due to missing data (only available for 66 (53%) of cases) and its minimal impact on effect size of other predictors (< 10% effect modification), leaving PaO2:FiO2 ratio as the only independent predictor for 30-day mortality (aHR 1.3; 95% CI, 1.01 to 1.6; P = 0.039 for every 50 mmHg decrease from the mean PaO2:FiO2 ratio). Concomitant antituberculosis therapy was also associated with a trend towards lower 30-day survival (aHR 1.9; 95% CI, 0.91 to 4.0; P = 0.085). The proportional hazards assumption was confirmed for the model (global test P-value = 0.168).

**Fig 3:**
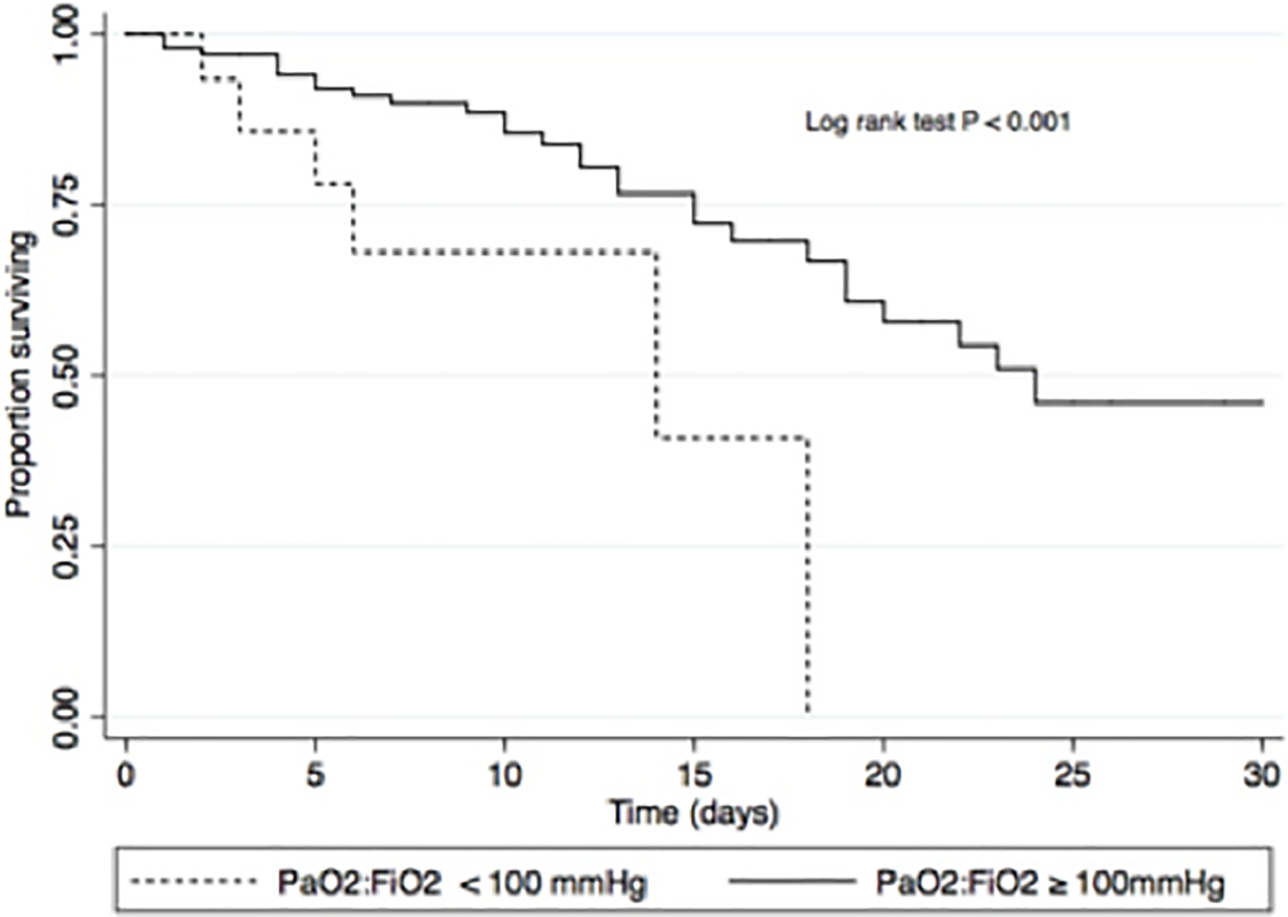
Survivor function adjusted for concomitant TB therapy.

Categories for PaO2:FiO2 ratio were generated to reflect clinically-relevant predictors for risk-stratification. The number of inpatient deaths amongst patients with severe acute respiratory distress syndrome (ARDS, PaO2:FiO2 < 100 mmHg) was significantly higher than those with less severe forms PaO2:FiO2 ≥ 100 mmHg (7/9 deaths (78.8%) vs. 28/91 deaths (30.8%); P = 0.005), and this remained an independent predictor when substituted for the continuous variable in the multivariate model (HR 3.8; 95% CI, 1.6 to 8.9; P = 0.003). To illustrate this effect, the adjusted survivor function, stratified by the presence of severe ARDS, is shown in Fig 3.

## Discussion

Although PCP is increasingly recognized as an important cause of pneumonia amongst HIV-infected adults in sub-Saharan Africa [1, 2], there is very limited knowledge on its clinical phenotype and outcomes in this population [1]. We have shown in this retrospective cohort study that HIV-associated PCP is associated with a high mortality amongst inpatients at a South African hospital and have identified severe impairment of oxygenation, serum LDH, and concurrent antituberculosis therapy as key factors associated with poor outcomes.

To our knowledge, there have only been three studies (two from South Africa and one from Zimbabwe) that have specifically evaluated the clinical aspects and reported outcomes of HIV-associated PCP in Southern Africa [13, 21, 22]. These studies were all published over 17 years ago, before the widespread introduction of ART in the region; they had smaller sample sizes, and did not include patients admitted to ICU. Their reported inpatient mortality ranged between 10 to 15%, lower than what was observed in our cohort, which may have included sicker patients. In a recent systematic review of HIV-associated PCP in sub-Saharan Africa[2] the inpatient case fatality rate across 12 studies (including 334 patients) was 18.8%, similar to our finding (18.3%). This appears to be higher than in well-resourced settings. For example, in a large retrospective cohort (n = 494) from London hospitals the 4-week mortality rate for inpatients with HIV-associated PCP was 13.5% (with 11% of cases requiring ICU admission)[23]. The profile of patients with PCP at our center reflects a persistent subpopulation of the HIV epidemic that do not access ART and present late with low CD4 counts [24], and had unique characteristics that may contribute to worse outcomes. These include a high proportion of co-infections with tuberculosis (23.6%) and other bacterial pathogens (14.3%), very low median CD4 cell counts (26 cells/mm^3^, IQR 12 to 70), and severe impairment of oxygenation (median PaO2 6.9 kPa, IQR 5.7 to 7.9) at presentation.

Unsurprisingly, both inpatient and 90-day mortality was higher in those admitted to ICU. This was largely accounted for by selection of sicker patients: those in ICU had worse indices of oxygenation and inflammatory markers, and more rapid progression of disease from symptom onset. The early (57%) and late (62%) mortality of ICU patients in our cohort are similar to that reported in contemporary studies from high-income countries [8, 10–12]. This is despite our ICU patients having high rates of TB co-infection and being particularly ill, with a median PaO2:FiO2 ratio of 158 mmHg (representing moderately severe ARDS, which has been associated with an all-cause mortality of 32%) [25]. There are very scarce data on ICU outcomes of HIV-associated PCP in resource-limited countries, and none from Africa. One study from Thailand, involving 14 HIV-infected patients with PCP, reported a mortality of 57%, very similar to our experience [16]. However, direct comparisons between studies have limited value due to heterogeneity in severity of illness and management strategies.

Many studies have attempted to identify prognostic factors for HIV-associated PCP, with inconsistent findings. We attempted to broadly capture these factors for both early and late mortality in our univariate analysis because the clinical features of PCP have not been well-characterized in African populations. Oxygenation index (PaO2:FiO2 ratio), serum LDH, and concurrent antituberculosis therapy were found to be independent predictors of mortality in our cohort, and showed reasonable performance on an internally generated validation dataset.

The association between concurrent tuberculosis and PCP mortality is a novel finding, and is relevant in settings such as southern Africa where there is a high burden of both these infections [2]. It is likely that patients with PCP-tuberculosis co-infection have more severe illness and other unexplored clinical confounders that account for the association with mortality, but it is also possible that an underlying pathophysiological interaction exists that may influence the disease course and outcomes of PCP [21].

A number of studies have identified an association between serum LDH levels and PCP prognosis [19, 26–28]. One of these demonstrated a negative correlation between LDH levels and oxygenation impairment (PaO2:FiO2) [27], suggesting this is likely to reflect underlying lung inflammation rather than a specific pathophysiological feature of the infection [29]. The combination of elevated serum LDH and oxygenation index, as seen in our study, have also been shown to predict mortality in other settings [26].

Impairment of gas exchange has been consistently identified as a risk factor for PCP-related death [6, 12, 16, 17, 23], and this is biologically plausible given the pathophysiology of PCP [30] and strong correlation between degree of oxygenation impairment and mortality risk in general [25]. We have showed that after adjustment, the PaO2:FiO2 ratio, and particularly severe ARDS, remains a powerful predictor of mortality and could be used to risk-stratify PCP patients for ICU admission in our setting.

Our study had important limitations. Although we conducted a systematic search for cases and undertook careful data collection with pre-specified variables, the retrospective study design results in missing data, and introduces bias in case selection, outcome ascertainment, and confounding in prediction models. Because we searched for potential cases using laboratory requests, we may have missed a number of ‘probable PCP’ patients during the study period who were unable to produce a sputum sample; this may be associated with a distinct disease phenotype which was not accounted for in our population. The ICU cases were highly selected based on the restrictive local admission criteria for PCP, and they may represent a patient group with specific clinical or radiological presentations that may have biased their outcomes. On the other hand, there may have been a number of ward patients who were denied ICU admission after the development of treatment failure, preventing an analysis of ICU outcomes in this subgroup. Although this is likely the largest study to provide a detailed clinical description of PCP in sub-Saharan Africa to date, our relatively small sample size and number of outcomes events restricted the number of variables included in regression models and limited their precision and predictive power. The model performed reasonably well on an internal validation sample but will need to be evaluated on an external population to properly assess the clinical usefulness of the predictors.

## Conclusions

Notwithstanding the above limitations, we have shown that the in-hospital and 90-day mortality of HIV-associated PCP amongst South African inpatients was high and was associated with a severe clinical phenotype and high rates of tuberculosis co-infection. Outcomes were particularly poor for patients admitted to the ICU for mechanical ventilation but are comparable to high-income settings. Tuberculosis co-infection, serum LDH, and PaO2:FiO2, were independent predictors of short and longer-term outcomes and could inform local ICU admission policy and criteria for mechanical ventilation for HIV-associated PCP. This serious opportunistic infection continues to afflict an important subgroup of people with advanced HIV in sub-Saharan Africa, and prospective cohort studies should be performed to more accurately determine prognostic markers in this understudied population.
